# Emerging Role of miRNAs in the Drug Resistance of Gastric Cancer

**DOI:** 10.3390/ijms17030424

**Published:** 2016-03-22

**Authors:** Ismael Riquelme, Pablo Letelier, Angela L. Riffo-Campos, Priscilla Brebi, Juan Carlos Roa

**Affiliations:** 1 Molecular Pathology Laboratory, Department of Pathology, CEGIN-BIOREN, Universidad de La Frontera, Avenida Alemania 0458, 3th Floor, Temuco 4810296, Chile; ismael.riquelme.contreras@gmail.com (I.R.); a.riffo.c@gmail.com (A.L.R.-C.); brebi.mieville@gmail.com (P.B.); 2 School of Health Sciences, Universidad Católica de Temuco, Manuel Montt 56, Temuco 4813302, Chile; pablolete@gmail.com; 3 Department of Pathology, UC Centre for Investigational Oncology (CITO), Advanced Centre for Chronic Diseases (ACCDiS), Pontificia Universidad Católica de Chile, Marcoleta 377, 7th Floor, Santiago 8330024, Chile

**Keywords:** microRNAs, drug resistance, gastric cancer

## Abstract

Gastric cancer is the third leading cause of cancer mortality worldwide. Unfortunately, most gastric cancer cases are diagnosed in an advanced, non-curable stage and with a limited response to chemotherapy. Drug resistance is one of the most important causes of therapy failure in gastric cancer patients. Although the mechanisms of drug resistance have been broadly studied, the regulation of these mechanisms has not been completely understood. Accumulating evidence has recently highlighted the role of microRNAs in the development and maintenance of drug resistance due to their regulatory features in specific genes involved in the chemoresistant phenotype of malignancies, including gastric cancer. This review summarizes the current knowledge about the miRNAs’ characteristics, their regulation of the genes involved in chemoresistance and their potential as targeted therapies for personalized treatment in resistant gastric cancer.

## 1. Introduction

Gastric cancer (GC) is the fifth most frequently-diagnosed cancer and the third most lethal malignancy worldwide, constituting an important public health problem. Each year, almost one million new GC cases are diagnosed, and ~700,000 people die of this disease, thus representing ~10% of the cancer-related deaths in the world [1]. GC prognosis and survival are highly dependent on the disease stage at diagnosis. Its high mortality rate is associated with a lack of validated screening programs and the absence of significant symptoms at early stages [2]. Therefore, most GC cases are diagnosed at an advanced stage, with a poor prognosis due to the limited efficacy of chemotherapy. Although many novel chemotherapeutic drugs are used in clinical practice, drug resistance is one of the leading causes of chemotherapy failure. Previous studies have clarified several cytological mechanisms of drug resistance of cancer cells, such as increased efflux of hydrophobic drugs, decreased uptake of water-soluble drugs and other changes influencing the capacity of cytotoxic drugs to kill cells, including alterations in cell cycle and proliferation, enhanced DNA repair activity, defective apoptosis, altered metabolism of drugs, and so forth [3,4,5,6]. Multiple paths could produce a drug resistance phenotype of cancer cells, such as genetic alterations, including mutations, translocations, deletions and amplification of genes or promoter regions, as well as epigenetic modifications. Recent studies have also reported that epigenetic mechanisms do not necessarily require a stable heritable genetic alteration and might play a key role in acquired drug resistance of cancer cells, which is highly relevant to clinical practice [7]. These epigenetic mechanisms include aberrant DNA methylation, histone modifications, non-coding RNA expression, *etc.* [8,9].

Advances in high-throughput technologies have led to the discovery of new transcripts in several types of diseases, including cancer. During the last decade, microRNAs (miRNAs), a group of small non-coding RNAs of ~21 nucleotides, have emerged as gene expression regulators of many normal and pathological cellular processes, even drug resistance, and have been proven particularly attractive targets to study in malignancies.

## 2. MicroRNAs: Biogenesis, Biological Role and Involvement in Drug Resistance

MicroRNAs (miRNAs) are non-coding RNAs of 19–25 nucleotides (~22 nt) that act as post-transcriptional regulators binding to the 3′ untranslated region (UTR) of target mRNA, specifically in the MRE (miRNA recognition element) sequence, in order to avoid the translation of this target mRNA, and, thus, regulate many homeostatic and pathological processes within cells [10,11,12,13]. The miRNA genes are usually transcribed by RNA polymerase II or III, generating an initial structure, a primary-miRNAs (pri-miRNA) that possesses a stem-loop hairpin structure of ~80 nts [14,15,16]. Mature miRNAs result from the cleavage of pri-miRNAs by the Drosha/DGCR8 complex (“microprocessor” complex) to form precursor miRNAs (premiRNA) of a ~60-nts hairpin [17]. Then, Exportin 5 (XPO5) and Ran-GTP export this pre-miRNA to the cytoplasm to be cleaved by the Dicer/TRBP complex, generating a miRNA/miRNA* duplex [18,19]. Finally, one strand of this miRNA duplex binds to the RNA-induced silencing complex (RISC), which carries this strand to the target mRNAs, whereas the other strand (miRNA* strand) is generally degraded [20,21,22] or can act as a regulatory mature miRNA [23]. Another miRNA biogenesis pathway involves short introns containing miRNA precursors that lack a stem-loop, called “mirtrons”. These miRNA precursors are digested via spliceosome [23,24] and processed in a Drosha- or Dicer-independent manner. Other miRNAs can be generated from an unusual hairpin structure processed by Ago2 instead of Dicer [25]. Regarding the miRNA biological role, these molecules are important in various biological processes, such as differentiation, proliferation and apoptosis [12,26], and can control multiple genes involved in cancer. The same miRNA can act as a tumor suppressor or as an oncogenic miRNA [27,28] due to tissue specificity characteristics. Oncogenic miRNAs (oncomiRs) act directly on mRNAs from genes with pro-apoptotic or anti-proliferative roles. Conversely, tumor-suppressor miRNAs repress the expression of genes with oncogenic functions.

**Figure 1:**
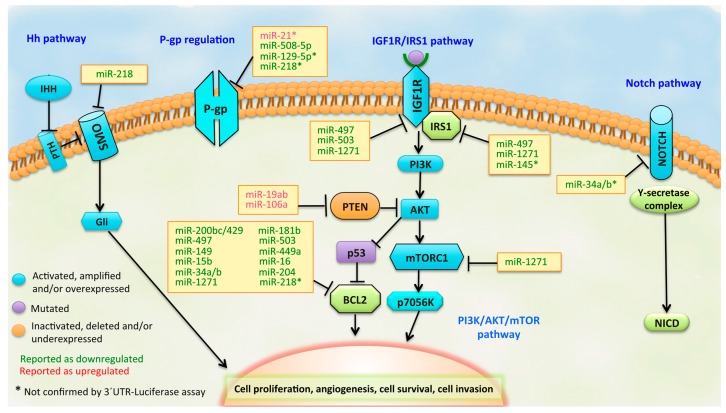
Pathways that represent potential targets for miRNAs in drug-resistant GC. The components of each signaling pathway are colored according to their dominant alteration type (see the key at the lower left). In the yellow boxes, the miRNAs are listed, including those downregulated (green letters) and those upregulated (red letters) in GC.

The expression pattern of miRNAs seems to have a critical role in drug resistance. More and more studies are demonstrating the importance of miRNAs in drug metabolism and disposition via the regulation of drug-metabolizing enzymes, drug transporters, transcription factor or nuclear receptors, which may not only provide insight into miRNA biological functions, but also advance the understanding of the integrated response of cells to xenobiotics [29]. Therefore, using *in silico* platforms, potential interactions between these drug transporters, nuclear receptors, transcription factors or drug-metabolizing enzymes with complementary miRNAs can be predicted [29,30] in order to evaluate these interactions later in a biological model. For example, one of the most common forms of resistance to chemotherapy is caused by overexpression of the multidrug transporters, such as ABCB1/MDR1 (that encodes P-glycoprotein) and ABCG2 genes. The overexpression of these transporters, especially P-gp, renders cancer cells resistant to a broad range of structurally- and functionally-diverse chemotherapeutic drugs [31]. In fact, there are some miRNAs able to induce changes in P-gp expression in various malignancies, including GC. Several miRNAs have been found to regulate the phenomenon of multidrug resistance in GC cells by exerting action in different signaling pathways (Table 1; Figure 1).

## 3. MicroRNAs as Regulators of Drug Resistance Pathways in GC

### 3.1. BCL2 Pathway

The *BCL2* (B-cell lymphoma-2) gene is located in chromosome band 18q21.3 and is part of the BCL-2 family of proteins that control the apoptosis process within cells. BCL-2 family members have been classified into three groups: anti-apoptotic proteins (BCL-2, BCL-XL, BCL-W, MCL1, BCL-B and BCL-2A1), pro-apoptotic proteins (BAX, BAK and BOK) and those proteins with BH3 domains that can bind and regulate the anti-apoptotic BCL-2 proteins to promote apoptosis (BAD, BIK, BID, HrK, BIM, BMF, NOXA and PuMA) [61]. As the *BCL2* gene encodes the anti-apoptotic protein BCL-2, the regulation in the expression of this target could be important in inducing apoptosis in cancer cells. Reports have stated that BCL-2 is a key regulator in chemotherapy resistance in several cancers, including GC [62,63].

In GC, several types of miRNAs have been implicated in the regulation of *BCL2* expression. For instance, the miR-200bc/429 cluster has been found to be downregulated, whereas *BCL2* and *XIAP* (X-linked inhibitor of apoptosis protein) have been found to be upregulated in the multidrug-resistant SGC7901/vincristine (VCR), compared to the parental SGC7901 cells. When the overexpression of the miR-200bc/429 cluster was induced, SGC7901/VCR showed an enhanced sensitivity to VCR, cisplatin (CDDP), etoposide (VP-16) and adriamycin (ADR), but not to 5-fluorouracil (5-Fu) compared to the miRNA mimic control-transfected cells. Meanwhile, those SGC7901 cells transfected with miR-200bc/429 cluster inhibitors exhibited greatly enhanced resistance to VCR, CDDP, VP-16 and ADR, but not to 5-Fu compared to the miRNA inhibitor transfected cells. Luciferase assays with both *BCL2* and *XIAP* 3′-UTR reporters constructed in resistant SGC7901 cells suggested that BCL2 and XIAP were the common target genes of the miR-200bc/429 cluster. This observation was also confirmed in Western blots by a reduced expression of BCL-2 and XIAP protein level in mimic-treated cells [32]. Similarly, miR-181b and miR-497 were also found to be downregulated in multidrug-resistant SGC7901/VCR cells, and this miR-181b downregulation correlated with the upregulation of BCL-2 protein, compared to the parental SGC7901 cells. An *in vitro* drug sensitivity assay demonstrated that overexpression of miR-181b sensitized SGC7901/VCR cells to anticancer drugs VCR, CDDP, 5-Fu, VP-16 and ADR, but not to mitomycin C (MMC). In the case of miR-497, those SGC7901/VCR cells transfected with miR-497 mimic exhibited greatly decreased resistance to VCR, CDDP, VP-16 and ADR, but not to 5-Fu compared to the miRNA mimic control transfected cells, while parental SGC7901 cells transfected with miR-497 inhibitor exhibited greatly enhanced resistance to VCR, CDDP, VP-16 and ADR, but not to 5-Fu compared to the miRNA inhibitor-transfected cells. The luciferase assay with a *BCL2* 3′-UTR-based reporter construct suggests that the *BCL2* gene is a target for both miR-181b and miR-497. Ectopic expressions of miR-181b and miR-497 reduced BCL-2 protein level and sensitized SGC7901/VCR cells to VCR-induced apoptosis [33,34]. Taken together, these data indicate that the miR-200bc/429 cluster and miR-181b could play a role in the development of multidrug resistance in GC cell lines, at least in part, through the modulation of apoptosis by targeting *BCL2* and *XIAP* in the case of miR-200bc/429 cluster or targeting only *BCL2* in the case of miR-181b and miR-497.

SGC7901/VCR and SGC7901/CDDP models have been also useful in determining the role of miR-503 in GC resistance. miR-503 was found downregulated in endoscopic GC tissues samples compared to non-tumor specimens. Furthermore, miR-503 was repressed in SGC7901/VCR and SGC7901/CDDP cell lines, while the expressions of *BCL2* and insulin-like growth factor 1 receptor (*IGF1R*) were upregulated in both cells, compared to the parental SGC7901 cells. Ectopic overexpression of miR-503 sensitized SGC7901/VCR and SGC7901/CDDP cells to VCR and CDDP, respectively. The corresponding luciferase assays confirmed the direct targeting of miR-503 on the mRNAs of *BCL2* and *IGF1R*, respectively. Enforced miR-503 expression decreased the BCL-2 protein level and sensitized both drug resistant cells to VCR-induced and CDDP-induced apoptosis, respectively [36,37]. Similar results were obtained for miR-143 in SGC7901/CDDP cells. The repression of miR-143 in GC tissues and cell lines agreed with the concurrent upregulation of *IGF1R* and *BCL2* in resistant cells compared to the parental SGC7901 cells. Treatment with miR-143 mimics sensitized SGC7901/CDDP cells to CDDP, and then, luciferase activity suggested that *IGF1R* and *BCL2* were both target genes of miR-143 [38].

Other studies have focused on miR-449a as a mediator of cell proliferation and chemosensitivity in GC cells via regulating cyclin D1 and BCL-2. The expression of miR-449a was downregulated in GC cell line SGC7901 and GC tissues compared to the gastric epithelial cell line GES-1 and matched non-tumor associated tissues. Ectopic upregulation of miR-449a reduced the proliferation of SGC7901 cells, decreased the percentage of S phase cells, increased the percentage of G1/G0 phase cells and increased the CDDP-induced apoptosis. This effect could be exerted by downregulating the translation of *BCL2* and *CCND1* mRNAs [40].

On the other hand, miR-15b and miR-16, members of the miR-15/16 family, have also shown a low expression in resistant SGC7901 cells. An MTT assay revealed that SGC7901/VCR cells transfected with miR-15b or miR-16 precursor exhibited greatly enhanced sensitivity to VCR, ADR, VP-16 and CDDP, but not to 5-Fu and MMC compared to those transfected with control oligonucleotides. Moreover, overexpression of miR-15b or miR-16 could sensitize SGC7901/VCR cells to VCR-induced apoptosis. Conversely, the suppression of the miR-15b or miR-16 level by transfecting with their specific inhibitors led to decreased sensitivity of SGC7901 cells to VCR, ADR, VP-16 and CDDP, conferring new multidrug-resistance on SGC7901 cells. As the downregulation of miR-15b and miR-16 in SGC7901/VCR cells was concurrent with the upregulation of BCL-2 protein, a *BCL2* 3′-UTR-based reporter assay was performed by transfecting miR-15b or miR-16 mimics into SGC7901/VCR cells. The results suggested that *BCL2* is a direct target of miR-15b and miR-16, which was supported by a reduced expression of BCL-2 protein level in Western blots. This indicates that miR-15b and miR-16 could play a role in the development of multidrug resistance in GC cells, at least in part through the modulation of apoptosis by targeting *BCL2* [41].

These miRNAs related to chemoresistance in GC have also been studied in other cell models. For instance, the effects of miR-34 restoration have been examined on p53-mutant human GC cells to evaluate its potential target gene expression. Kato III cells transfected either with miR-34 mimics or miR-34 lentiviral vector showed a reduced expression of target *BCL2*, *NOTCH1/2/3/4* and the high mobility group AT-Hook 2 (*HMGA2*) genes. Then, a luciferase assay confirmed that the *BCL2* gene is a direct target of miR-34, and more importantly, miR-34 restoration sensitized Kato III cells with a high level of BCL-2, but not MKN-45 cells with a low level of BCL-2. Finally, *in vitro* studies showed that miR-34 arrested cell growth, accumulated the cells in the G1 phase, increased caspase-3 activation and, more significantly, inhibited tumorsphere formation and growth. These data suggest that miR-34 restoration may regain P53 function and inhibit tumorsphere formation and growth, which correlates with the self-renewal features of cancer stem cells. Therefore, the possible mechanism of miR-34-mediated suppression could involve GC stem cell self-renewal/differentiation through downstream targets *BCL2*, *HMGA2* and NOTCH pathway members [42].

This relationship between aberrantly expressed miRNAs and the BCL-2 pathway has not only been described in resistant GC cell lines, but also in GC tissues. In 2012, Sacconi *et al.* [43] aimed to identify miRs whose deregulated expression leads to the activation of oncogenic pathways in GC. They analyzed 39 GC samples, and their matched uninvolved peritumoral gastric specimens from three independent European subsets of patients were analyzed for the expression of 851 human miRs using a high-throughput platform. Another 84 samples were used to validate miRs differentially expressed between tumor and matched peritumoral specimens by qPCR. miR-204 falls into a group of eight miRs differentially expressed between tumor and peritumoral samples. Downregulation of miR-204 had a prognostic value and correlated with increased staining of BCL-2 protein in tumor specimens. Complementary *in vitro* assays showed that miR-204 targeted *BCL2* mRNA and reduced BCL-2 protein expression in N87 and GTL-16 cell lines, which were correlated with Ki-67 expression. Moreover, miR-204 mimic transfection increased the responsiveness of these GC cells to 5-Fu and L-OHP (oxaliplatin) treatment. Ectopic expression of miR-204 significantly inhibited colony-forming ability, migration in N87 and GTL-16 GC cell lines and tumor engraftment of GTL-16 cells injected into CD1 mice. Conversely, ectopic expression of BCL-2 protein counteracted miR-204 pro-apoptotic activity in response to 5-Fu. Altogether, these findings suggest that modulation of aberrant expression of miR-204, which in turn releases oncogenic BCL-2 protein activity, might hold promise for preventive and therapeutic strategies for GC [43].

### 3.2. PI3K/PTEN/AKT Pathway

The importance of the physiological function of the phosphatase and tensin homologue (PTEN) is highlighted by its frequent deregulation in cancer, including GC [64,65]. Physiologically, PTEN suppresses the phosphoinositide 3-kinase (PI3K)/AKT/mammalian target of rapamycin (mTOR) pathway through its lipid phosphatase activity, thus governing several cellular processes, including survival, proliferation, energy metabolism, cellular architecture and cell sensitization to drugs, among others [66]. The activation of the PI3K pathway in cancers, by PTEN loss, reduction in PTEN expression or PI3K mutation, predicts lack of response to chemotherapy. Therefore, the status of the PTEN/PI3K pathway is significantly correlated with poor outcome from targeted therapy. Despite the signaling mechanisms responsible for the requirement of PTEN in targeted therapy efficacy being currently under rigorous investigation, it is known that PTEN somehow affects the action of targeted chemotherapy [67]. The deregulation of PTEN can be exerted by various molecular mechanisms, including genetic mutations, epigenetic silencing, transcriptional repression, post-translational modifications, the aberrant localization of PTEN, disruption of competitive endogenous RNA (ceRNA) networks and deregulation by microRNA (miRNA) expression changes [66].

For instance, miR-19a/b, a member of the miR-17-92 cluster, is considered an oncomiR and can influence multiple aspects of the malignant phenotype of GC. miR-19a/b was found upregulated in multidrug-resistant GC cell lines (SGC7901/VCR and SGC7901/ADR cells), and MTT assays revealed that SGC7901 cells transfected with the miR-19a/b mimic exhibited greatly decreased sensitivity to CDDP, 5-Fu and ADR. On the other hand, the suppression of the miR-19a/b level in SGC7901/VCR cells led to an enhanced sensitivity to CDDP, 5-Fu and ADR. Then, miR-19a/b was shown to accelerate the ADR efflux of GC cells by increasing the levels of *ABCB1* gene (P-gp protein) and to suppress drug-induced apoptosis by regulating BCL-2 and BAX. However, this was only a downstream effect, because, finally, *PTEN* mRNA was ultimately identified as the functional target of miR-19a/b [44]. Recent studies have shown that miR-106a is also overexpressed in GC and contributes to tumor growth and drug resistance. The SGC7901/CDDP cells showed higher levels of miR-106a expression compared to parental SGC7901 cells, and transfection with a miR-106a mimic induced resistance to CDDP in wild-type SGC7901. Conversely, suppression of miR-106a in SGC7901/CDDP led to enhanced CDDP cytotoxicity. There was a strong inverse correlation between the miR-106a and PTEN levels, and computational analyses predicted that *PTEN* was a conserved target gene of miR-106a. Thus, a luciferase reporter assay was performed, which confirmed that *PTEN* is the target gene of miR-106a. This result was confirmed by the downregulation of *PTEN* expression at mRNA and protein levels in SGC7901 transfected with a miR-106a mimic. Therefore, overexpression of miR-106a activates the PI3K/AKT pathway through its inhibitory role on PTEN [45].

Another example is miR-21, which was found to be upregulated in the CDDP-resistant cell line SGC7901/CDDP compared to its parental line SGC7901 line. When miR-21 was overexpressed, it significantly decreased the antiproliferative effects and apoptosis induced by CDDP, whereas knockdown of miR-21 dramatically increased the antiproliferative effects and CDDP-related apoptosis. In addition, miR-21 induced cell survival and CDDP resistance through direct downregulation of PTEN and activation of the PI3K/AKT pathway. When AKT was inhibited using the PI3K inhibitor, LY-294002, was able to abrogate miR-21-induced cell survival, which suggests that miR-21 may provide a novel mechanism for understanding resistance to CDDP in GC involving the PI3K/PTEN/AKT pathway [47]. Later, the Trastuzumab for Gastric Cancer (ToGA) clinical trial demonstrated the significant efficacy of trastuzumab in addition to chemotherapy in patients with HER2-positive GC; however, resistance to trastuzumab is a major problem in clinical practice. Therefore, Eto *et al.* [48] endeavored to identify a miRNA/gene pathway able to regulate the sensitivity of HER2-positive GC cells to trastuzumab. They evaluated miR-21 expression levels in three HER2-positive GC cell lines (MKN45, NUGC4, NCI-N87). Of these cell lines, miR-21 had a high expression in NUGC4 and a low expression in NCI-N87 cells. They found that overexpression of miR-21 in NCI-N87 cells downregulated PTEN expression, increased p-AKT and did not affect HER2 expression. The opposite effect in PTEN and p-AKT was observed in NUGC4 cells after miR-21 suppression. Ectopic overexpression of miR-21 decreased the sensitivity of GC cells to trastuzumab and trastuzumab-induced apoptosis, while miR-21 inhibitors restored such resistance. These data suggest that the miR-21/PTEN pathway potentially regulates the sensitivity of HER2-positive GC cell lines to trastuzumab through modulation of apoptosis, which may lead to the development of individualized treatment in clinical practice [48].

### 3.3. IGF1R/IRS1 Pathway

The insulin-like growth factor 1 receptor (IGF1R)/IRS1 pathway is critical to cellular proliferation, apoptosis and interactions with the microenvironment. The IGF system is comprised of two ligands, IGF-1 and IGF-2, which exhibit their effects through binding to IGF1R (primarily), IGF2R and the insulin receptor (IR), all belonging to the tyrosine kinase receptor family. Upon binding the IGF ligand, IGF1R is activated through autophosphorylation and subsequently phosphorylates insulin receptor substrate 1 (IRS1). Then, PI3K/AKT/mTOR and Ras/MAPK signaling pathways are activated in parallel. Particularly, phosphorylated AKT (p-AKT) performs a variety of functions, such as releasing the anti-apoptotic protein BCL-2 from BAD, activating protein synthesis through mTOR and promoting glucose metabolism by inhibiting GSK-3b [68]. Therefore, the IGF1R/IRS1 pathway is commonly referred to as a part of the PI3K/AKT pathway because the latter is ultimately responsible for preventing cell death. Of the many processes that are thought to play a role in the resistance of neoplasms to radiation or chemotherapy, the IGF signaling axis has been recurrently deemed the culprit [69].

Some miRNAs have been involved in IGF1R/IRS1-pathway-induced chemoresistance in GC. For example, miR-1271 has been studied as a possible inductor of CDDP resistance in GC cells. miR-1271 was found to be significantly downregulated in GC tissues and cell lines. More interestingly, this downregulation was even lower in the SGC7901/CDDP cells and was accompanied by the upregulation of IGF1R/IRS1 pathway-related proteins, *i.e.*, IGF1R, IRS1, mTOR and BCL-2 in these cells compared to the parental SGC7901 cells. Ectopic expression of miR-1271 sensitized SGC7901/CDDP cells to CDDP, and the luciferase assay with 3′-UTR reporter constructs of the above-mentioned proteins in SGC7901/CDDP cells suggested that *IGF1R*, *IRS1*, *MTOR* and *BCL2* genes are targets of miR-1271. Transfection with miR-1271 mimics repressed the protein levels of its targets, inhibited proliferation of SGC7901/DDP cells and sensitized SGC7901/DDP cells to CDDP-induced apoptosis. These data proposed that miR-1271 could regulate CDDP resistance in GC cells, at least partially, via targeting the IGF1R/IRS1 pathway [50].

Another example is miR-497, which was previously found downregulated in GC and linked to BCL2 expression in GC cells resistant to VCR, CDDP, VP-16 and ADR [34]. In addition, He *et al.* [35] found that the resistance to CDDP was not only related to the miR-497 action on *BCL2* mRNA, but also the action of miR-497 on *IGF1R* and *IRS1* mRNAs in SGC7901/CDDP cells, suggesting this miRNA could modulate CDDP resistance of GC cells in part by targeting the IGF1R/IRS1 pathway [35].

Similarly, miR-143 and miR-145 have also been associated with resistant GC. Levels of miR-143 and miR-145 were reduced in most of the GC tissue samples examined. Treatment with miR-145 mimic in MKN-1 and KATO III cells resulted in a greater growth inhibitory effect than that induced by miR-143; however, an additive effect on growth inhibition was shown by the combined transfection with miR-143 and miR-145 in MKN-1 cells. Moreover, a higher sensitivity to 5-Fu was also observed following the transfection with miR-143 or miR-145. Transfection with miR-143 reduced levels of ERK5 protein in Western blot, but not *ERK5* mRNA levels. *In silico* analyses suggested that miR-143 possibly targets the 3′-UTR region of *AKT1*, which was confirmed by the downregulation of AKT at a translational level. On the other hand, computational analyses showed that *IRS1* and *ACTB* (β-actin) mRNAs are possible candidate targets of miR-145, which was ratified by a reduction of both proteins in Western blot and a slightly decreased level of *IRS1* mRNA [39].

### 3.4. ABCB1 (MDR1/P-gp) Regulation

*ABCB1*, also known as *MDR*, is a gene belonging to the ubiquitous adenosine triphosphate (ATP)-binding cassette (ABC) family that encodes a transporter and channel protein named glucoprotein-P (P-gp) that has a membrane-spanning domain, which forms a pore and possesses an intracellular nucleotide-binding domain for the ATP-dependent translocation of substrates or ions across the cell membrane. P-gp has protective and excretory functions and plays an important role in the first-pass elimination of drugs to limit their bioavailability by effluxing drugs from certain cells [70]. In this regard, P-gp overexpression has been associated with the development of multidrug resistance of cultured tumor cells against various anticancer agents. However, this transporter is not only expressed in tumor cells, but also in normal tissues with excretory function (intestine, liver, kidney) [71]. Numerous common coding variants in *ABCB1* have been studied for their potential influence on P-gp expression, function and disease risk. However, genetic associations with molecular or clinical phenotypes have largely been inconsistent [71,72,73]. As a result, epigenetic modifications, including changes in miRNA patterns, are now being studied in order to elucidate the control mechanisms for *ABCB1* expression.

In this context, miR-21 was studied to evaluate its involvement in the development of resistance to paclitaxel (PTX) in GC cells. The levels of miR-21 were found to be upregulated in SGC7901 cells resistant to PTX (SGC7901/PTX) compared to parental SGC7901 cells. Overexpression of miR-21 in SGC7901 induced significantly decreased antiproliferative effects and PTX-induced apoptosis, while the miR-21 knockdown dramatically induced the opposite effect. As the *ABCB1* gene was a candidate for miR-21 targeting, the SGC7901 and SGC7901/PTX cells were transfected with mimics and inhibitors of miR-21, respectively. Treatment with miR-21 mimics resulted in increased expression levels of P-gp in wild-type SGC7901 cells, whereas treatment with miR-21 inhibitors showed decreased levels of *ABCB1* mRNA and P-gp protein expression in the SGC7901/PTX cells. Therefore, given the modulating effect of miR-21 on PTX sensitivity, these results suggest that miR-21 might work via regulating somehow the P-gp expression, which is involved in PTX resistance in SGC7901/PTX cell lines [49].

Using high-throughput functional screening, Shang *et al.* [53] revealed a total of 11 miRNAs involved in multidrug resistant cell line SGC7901/VCR. The overexpression of miR-508-5p in SGC7901/VCR and SGC7901/ADR cells was sufficient to reverse cancer cell resistance to multiple chemotherapeutics (ADR, VCR, 5-Fu and CDDP) *in vitro* and to sensitize tumors to chemotherapy *in vivo*. Further studies showed that miR-508-5p could directly target the 3′-UTR regions of *ABCB1* and zinc ribbon domain-containing 1 (*ZNRD1*), suppressing their expression at mRNA and protein levels. Interestingly, when *ZNRD1* is repressed, a decrease in *ABCB1* is also induced. All of these findings suggest that this miR-508-5p/*ZNRD1*/*ABCB1* regulatory loop has a critical role in multidrug resistance in GC, and miR-508-5p could constitute a prognostic factor for drug resistance and overall survival in this malignancy [53]. Employing a similar approach, Zhang *et al.* [51] analyzed miRNA expression levels between multidrug-resistant SGC7901/ADR cells and parent SGC7901 cells using a microarray. Among the differentially-expressed miRNAs, miR-103/107 was notably downregulated in resistant cells. Overexpression of miR-103/107 induced sensitization in SGC7901/ADR cells to doxorubicin (DOX), as demonstrated by *in vitro* and *in vivo* drug sensitivity assays. Then, the authors further confirmed that miR-103/107 inhibited P-gp function in SGC7901/ADR cells as a consequence of its direct targeting on the caveolin-1 gene (*CAV1*) [51]. CAV1 is a critical component of caveolae (a special type of lipid raft) [74] and interacts with P-gp to modulate P-gp transport activity [75,76], which might indicate that caveolae represent the platform for P-gp channel formation.

Recent studies have reported that hypermethylation in the promoter region of miRNAs could silence the expression of tumor suppressor miRNAs. However, the potential mechanism regarding how methylation of a miRNA CpG island could regulate cancer cell chemoresistance is unclear. In this regard, Wu *et al.* [54] performed microarray and BSP (bisulfate sequencing PCR) assays and found that miR-129-5p was hypermethylated and downregulated in multiresistant SGC7901/VCR cells compared to parental cells. Then, this miR-129-5p expression was restored in SGC7901/VCR cells treated with a demethylation agent (5-Aza-dC). They also found that miR-129-5p overexpression reduced the chemoresistance of SGC7901/VCR and SGC7901/ADR cells to VCR, 5-Fu and CDDP, whereas miR-129-5p antagomiR had the opposite effect in SGC7901 cells *in vitro* and *in vivo*. Furthermore, using bioinformatics analysis and report gene assays, the authors found that three members of the ABC transporter genes (*ABCB1*, *ABCC5* and *ABCG1*) were direct targets of miR-129-5p. Therefore, hypermethylation of the miR-129-5p CpG island might play an important role in the development of GC chemoresistance by targeting key drug transporters in GC [54].

### 3.5. Other Signaling Pathways of Drug Resistance in GC

Some regulators belonging to other important signaling pathways (*i.e.*, Hedgehog, autophagy and cell cycle pathways, among others) have also been involved in miRNA-related chemoresistance within GC cells. For instance, the Hedgehog (Hh) signaling pathway, which is essential in cell differentiation, embryonic development and adult stem cell maintenance, has also been implicated due to deregulation of miR-218. Two multidrug-resistant GC cell lines—SGC7901/ADR and SGC7901/oxaliplatin (l-OHP) cells—showed lower expression of miR-218 compared to parental cells. Overexpression of miR-218 chemosensitized these GC cells to ADR, 5-Fu and l-OHP, accelerated drug-induced apoptosis and reduced the gene and protein expressions of P-gp and BCL-2. Interestingly, *in silico* analyses and luciferase assays confirmed that the smoothened (*SMO*) gene, a transmembrane protein and member of the Hh pathway, is a functional target of miR-218. More importantly, *SMO* overexpression counteracts the chemosensitizing effects of miR-218 in the above-mentioned cell models, becoming a promising target for GC multidrug resistance [58]. The autophagy pathway has also been implicated in GC chemoresistance. Using gain or loss-of-function experiments, An *et al.* [56] found that miR-23b-3p could be used as a prognostic factor for overall survival in GC. In fact, overexpression of miR-23b-3p reversed resistance to multiple chemotherapeutics *in vitro* and sensitized tumors to chemotherapy *in vivo*. This miRNA was described as a direct silencer of autophagy-specific gene 12 (*ATG12*) and the high mobility group box 2 (*HMGB2*) gene that were positively associated with the occurrence of autophagy. When the expression of these target genes was repressed by siRNA or by inhibition of autophagy, SGC7901 cells were sensitized to chemotherapy. In summary, miR-23b-3p inhibits autophagy mediated by ATG12 and HMGB2 proteins and sensitized GC cells to chemotherapeutic agents, such as VCR, CDDP and 5-Fu, playing a potential critical role in multidrug-resistant GC [56]. The cell cycle pathway has also been involved in chemoresistant GC. MiR-223 was found to be upregulated in GC tissues and in resistant cells (SGC7901/CDDP and BGC-823/CDDP) compared to matched non-tumor tissues and corresponding parental GC cells, respectively. The F-Box and WD repeat domain containing 7 (*FBXW7*) gene, which function in phosphorylation-dependent ubiquitination, was identified as the direct and functional target of miR-223. Overexpression of *FBXW7* could mimic the effect of miR-223 downregulation, and silencing of *FBXW7* could partially reverse the effect of miR-223 downregulation in CDDP-resistant GC cells. Inhibition of miR-223 and *FBXW7* overexpression could affect the G1/S transition of the cell cycle in SGC7901/CDDP lines by downregulating CDK2, CDK4, CDK6, CCND1, CCND2 and CCND3 and upregulating P14, P16, P21 and P27 cell cycle regulators. Furthermore, data from patients helped to infer that miR-223 was found to be significantly upregulated in *Helicobacter pylori*-infected tissues and cells, suggesting that an infection by *Helicobacter pylori* might be important in the development of CDDP-resistant GC [57]. Moreover, the MAPK, WNT and P53 signaling pathways have been involved in chemoresistance through the potential complementarity of miR-125b to different target genes within these pathways. This idea came from the fact that miR-125b is downregulated in 5-Fu-resistant BGC823 (BGC823/Fu) cells [55].

Another example is miR-23a, which was found to be significantly upregulated in human GC tissues [77]. Liu *et al.* confirmed this upregulation of miR-23a in GC tissues compared to gastric non-tumor samples [60]. Then, they demonstrated that miR-23a suppresses paclitaxel (PTX)-induced apoptosis, promotes cell proliferation and the colony-forming ability of MGC803 and BGC823 cells by directly targeting the interferon regulator factor 1 (*IRF1*) gene, an activator of α/β interferons and a regulator of apoptosis and tumor suppression. This was confirmed in GC tissues, where expression of miR-23a was frequently higher, whereas IRF1 was downregulated compared to gastric non-tumor tissues. Ectopic expression of *IRF1* markedly promoted PTX-induced apoptosis and inhibited cell viability and colony-forming ability, whereas the knockdown of IRF1 had the opposite effect. Furthermore, restoration of *IRF1* expression counteracted the above-mentioned effects of miR-23a on the PTX-induced apoptosis and cell proliferation of GC cells. These results show that miR-23a effectively blocks *IRF1* expression, subsequently inducing suppression in PTX-induced apoptosis and other key carcinogenic features [60].

Du *et al.* [52] investigated whether there was a correlation between miR-20a and the NFκB pathway in order to clarify the effects of miR-20a in GC chemoresistance. They found that miR-20a was significantly upregulated in GC plasma and tissue samples compared to the controls, but this upregulation was higher in plasma and tissues from patients with CDDP-resistant GC. In addition, miR-20a was found to be upregulated in SGC7901/CDDP cells compared to SGC7901 cells. Upregulation of miR-20a was concurrent with the downregulation of a key regulator in the NFκB pathway, the *NFKBIB* gene, which was further confirmed as a direct target gene of miR-20a by luciferase assays. Interestingly, miR20a upregulation also was concurrent to upregulation of p65, livin and survivin. Transfection of miR-20a inhibitor could increase *NFKBIB* levels, downregulate the expression of p65, livin and survivin and lead to a higher proportion of apoptotic cells in SGC7901/CDDP cells. Meanwhile, ectopic expression of miR-20a in SGC7901 cells has the opposite effect, in particular a decrease in the apoptosis induced by CDDP in these cells. Therefore, Du *et al.* suggested that miR-20a could promote chemoresistance via activation of the NFκB pathway and downstream targets, livin and survivin, induced by *NFKBIB* silencing [52].

Another report focused on the methylation status of neighboring CpG islands of miR-34c-5p and its involvement in chemoresistance in GC. The miR-34c-5p expression was found to be downregulated in PTX-resistant GC samples. Those cells derived from GC tissues with low miR-34c-5p expression and high microtubule-associated protein tau (MAPT) protein expression tended to have increased resistance to PTX *in vitro*. Interestingly, *MAPT* mRNA was shown to be a direct target of miR-34c-5p, which was confirmed by a decreased MAPT protein expression after overexpression of miR-34c-5p. Treatment with a miR-34c-5p mimic also increased the chemosensitivity of resistant SGC7901/VCR cells to PTX. Finally, it was demonstrated that differential methylation of CpG islands neighboring the miR-34c promoter regulated the expression of miR-34c-5p in GC cell lines, resulting in the deregulation of *MAPT* expression and ultimately provoking the PTX-resistant phenotype [59].

## 4. miRNA Signatures in Drug-Resistant GC

Some studies involving high-throughput technologies have been performed to investigate the involvement of miRNAs in the intrinsic drug resistance of GC. Wu *et al.* [78] studied the miRNA expression patterns and their potential mRNA targets in six GC cell lines (BGC-823, SGC-7901, MGC-803, HGC-27, NCI-N87 and AGS) resistant to hydroxycamptothecin (HCPT) by using microarrays. Gene ontology and pathway analysis was conducted using GenMAPP2. In the HCPT-resistant GC cells, the levels of 25 miRNAs were differentially expressed (*i.e.*, upregulated: let-7g, miR-19b, miR-132, miR-224, miR-338, miR-365, miR-424, miR-452, miR-98; and downregulated: miR-200a, miR-200b, miR-200c, miR-141, miR-429, miR-7, miR-31, miR-372, miR-373, among others). Moreover, 307 genes were differentially expressed in HCPT-resistant cell lines, including chemoresistance-related genes, such as *CDKN1B* (p27), *ANXA*1 (p35), *PDCD4*, *UGT1A1*, *TOP1*, *CYP3A4*, *ABCG2 (BCRP)*, *CHEK1*, *TDP1*, *BCL2* and *SUMO1*, and genes from drug metabolism-associated pathways (*i.e.*, *CYP450*, *etc.*). The hierarchical clustering showed that the miRNA and mRNA signatures in these results were informative for discriminating cell lines with different sensitivities to HCPT. However, there was slightly lower correlation between the expression patterns of the miRNA and those of the predicted target transcripts [78].

Similarly, Kim *et al.* [79] studied the miRNA signatures of GC resistant to CDDP and 5-Fu (CDDP/5-Fu) through miRNA microarray analysis using endoscopic biopsy samples collected prior to chemotherapy from 90 GC patients treated with CDDP/5-Fu, 34 healthy volunteers and from eight post-treatment responders. They identified a miRNA expression signature that distinguishes GC from the normal stomach epithelium of healthy volunteers. Among those upregulated miRNAs associated with chemosensitivity were let-7g, miR-342, miR-16, miR-181, miR-1 and miR-34. Interestingly, a panel of 58 miRNAs was identified as a predictor to effectively separate those pre- and post-treatment tumor samples from the eight clinical responders (low-risk category), whereas the same predictor panel could separate samples from the post-treatment tumors that developed chemoresistance as a high-risk group, suggesting that selection for the expression of these miRNAs occurred as chemoresistance arose. However, further research is needed to validate and characterize the functions of these miRNAs [79].

Huang *et al.* [80] identified five miRNAs (miR-1, miR-20a, miR-27a, miR-34a and miR-423-5p) that are upregulated in GC. They evaluated the value of these miRNAs as potential biomarkers for predicting chemosensitivity and prognosis in metastatic or recurrent GC patients who received first-line chemotherapy. Patients receiving first-line chemotherapy with fluoropyrimidine combined with oxaliplatin (l-OHP) or paclitaxel (PTX) were chosen for the chemosensitivity analysis. Patients with upregulated miR-27a expression had a significantly worse overall survival (OS) than patients with a lower miR-27a expression, constituting a potential biomarker for predicting resistance to fluoropyrimidine-based chemotherapy in patients with multidrug-resistant GC and a novel prognostic marker for GC [80].

In order to offer a future perspective of the use of these miRNAs in the therapy selection, Table 2 summarizes the potential treatment protocols for patients with CG based on the expression patterns of certain miRNAs.

## 5. Concluding Remarks

Drug resistance is one of the major complications of GC therapy, and alterations in miRNA expression patterns have been shown to contribute importantly to this resistance in GC. The regulation of certain miRNA expressions could partially improve the response of GC cell lines to chemotherapy and significantly enhance the antitumor properties of specific drugs. The knowledge of the emerging role of these miRNAs in drug resistance, either alone or in networks, is very helpful for developing personalized antitumor regimens, by predicting the potential resistance of cancer cells, as well as establishing novel therapeutic strategies to reverse the resistance of tumors in combination with chemotherapeutic agents. miRNA mimics and antagonists are single-stranded RNAs capable of imitating and silencing, respectively, the activity of a specific miRNA. However, these mimics or antagomirs have not yet been used to a large degree in clinical trials, despite being relatively safer in early preclinical trials compared to treatments based on RNA interference (RNAi), such as small interfering RNAs (siRNAs) or short hairpin RNAs (shRNAs), which have been found to be toxic in preclinical mouse models [81,82]. Although the toxicity-inducing mechanism is not well known, this toxicity might be caused by two main reasons: (1) a dose-dependent oversaturation of siRNAs or shRNAs within cells [83], likely because these molecules have a lower propensity to be processed by Pol-II compared to miRNA-based treatments [81]; (2) a disruption of normal cellular processes induced by siRNAs or shRNAs, but not by miRNAs analogs (*i.e.*, myotube elongation) [81]. In addition, the chemical and viral transfection vehicles are also toxic agents commonly used in both RNAi and miRNA-based strategies [82]. The effects of this toxicity involves a higher immune response in mice, which is mainly seen as tissue damage in some organs, such as liver, kidneys and brain, in these animals [81,82,84].

The only exception for miRNA-targeted treatment is miravirsen, which has been introduced in a phase II clinical trial for hepatitis C therapy to inhibit miR-122, the essential miRNA for hepatitis C virus replication [85]. This approach is very promising for successfully introducing small RNAs in anticancer treatments and opening the door for their future clinical use. Another rising strategy to solve gene specificity limitations is the technology of genome editing by clustered regulatory interspaced short palindromic repeats-associated endonuclease 9 (CRISPR-Cas9), which is a rapid and efficient way to generate total or partial downregulation of specific genes, including miRNAs, by the targeted interruption of the promoter and the chosen sequence through insertion of polyadenylation signals. Furthermore, CRISPR-Cas9 can be applied to achieve miRNA overexpression from its endogenous locus by inserting a strong promoter upstream of the miRNA sequence or by targeting transcriptional activator complexes to the promoter [86,87,88].

During the last decade, blood-circulating miRNAs have emerged as a potential tool in the screening, selection and follow-up of GC patients in order to supply a personalized treatment. These miRNAs can be released from tumor cells either in a cell-free form or within microvesicles, such as exosomes. The main advantages of this approach are the non-invasive sampling, a good correlation with tumor size, better detection sensitivity and a good stability in the bloodstream (especially those miRNAs transported within exosomes) compared to other cell-free nucleic acids [89,90,91]. Therefore, circulating miRNAs might be important for patients in order to select the drug protocol and then in the follow-up of treated patients for evaluating the effectiveness of these drug protocols.

Although there are still multiple challenges to overcome before miRNA therapeutics can be used clinically, including, but not limited to, chemical modification and delivery of miRNA regulators into tumors within patients, it is predicted that in the near future, miRNA-based approaches may provide important advances in overcoming drug resistance and improving chemotherapy response and quality of life in cancer patients. Further studies are needed to discover more miRNA targets and to acquire a better understanding of the mechanisms of multidrug resistance in GC.

## Figures and Tables

**Table 1:** MicroRNAs involved in drug resistance of gastric cancer (GC).

miRNA	Expression	Target Genes ^1^	Tested Drugs ^2^	References
miR-200bc/429	Down	*BCL2*, *XIAP*	VCR, CDDP, VP-16, ADR, 5-Fu	[32]
miR-181b	Down	*BCL2*	VCR, CDDP, 5-Fu, VP-16, ADR, MMC	[33]
miR-497	Down	*BCL2*, *IGF1R*, *IRS1*	VCR, CDDP, VP-16, ADR, 5-Fu	[34,35]
miR-503	Down	*BCL2*, *IGF1R*	VCR, CDDP	[36,37]
miR-143	Down	*BCL2*, *IGF1R*, ERK5 *	CDDP, 5-Fu	[38,39]
miR-449a	Down	*BCL2, CCDN1*	CDDP	[40]
miR-15b	Down	*BCL2*	VCR, CDDP, VP-16, ADR, 5-Fu, MMC	[41]
miR-16	Down	*BCL2*	VCR, CDDP, VP-16, ADR, 5-Fu, MMC	[41]
miR-34a/b	Down	BCL2 NOTCH pathway *, *HMGA2*	CDDP, DOX, DTX, GEM	[42]
miR-204	Down	*BCL2*	5-Fu, L-OHP	[43]
miR-19a/b	Up	*PTEN*	CDDP, 5-Fu, ADR	[44]
miR-106a	Up	*PTEN*, *RUNX3*	CDDP, ADR	[45,46]
miR-21	Up	*PTEN*, ABCB1 *	CDDP, PTX, Trastuzumab	[47,48,49]
miR-1271	Down	*IGF1R*, *IRS1*, *MTOR BCL2*	CDDP	[50]
miR-103/107	Down	*CAV1*	DOX	[51]
miR-20a	Up	*NFKBIB*	CDDP	[52]
miR-145	Down	IRS1 *, BACT *	5-Fu	[39]
miR-508-5p	Down	*ABCB1*, *ZNRD1*	VCR, CDDP, 5-Fu, ADR	[53]
miR-129-5p	Down	ABCB1 *, ABCC5 *, ABCG1 *	VCR, ADR, CDDP, 5-Fu	[54]
miR-125b	Down	MAPK pathway **, WNT pathway **, P53 pathway **	5-Fu	[55]
miR-23b-3p	Down	*ATG12*, *HMGB2*	VCR, CDDP, 5-Fu	[56]
miR-223	Up	*FBXW7*	CDDP	[57]
miR-218	Down	*SMO*, ABCB1 *, BCL2 *	ADR, 5- Fu, L-OHP	[58]
miR-34c-5p	Down	*MAPT*	PTX	[59]
miR-23a	Up	*IRF1*	PTX	[60]

^1^ ABCB1 (ATP-binding cassette, sub-family B, member 1), IGF1R (insulin-like growth factor 1 receptor), IRS1 (insulin receptor substrate 1), ZNRD1 (zinc ribbon domain-containing), PTEN (phosphatase and tension homolog), BCL2 (B-cell lymphoma 2), XIAP (X-linked inhibitor of apoptosis protein), RUNX3 (runt-related transcription factor 3), SMO (Smoothened), MAPT (microtubule associated protein tau), HMGA2 (high-mobility group AT-hook 2), IRF1 (interferon regulator factor 1); ^2^ VCR (vincristine), CDDP (cisplatin), ADR (adriamycin), VP-16 (etoposide), 5-Fu (5-fluoruracil), MMC (mitomycin C), DOX (doxorubicin), DTX (docetaxel), GEM (gemcitabine), PTX (paclitaxel), L-OHP (oxaliplatin); * not confirmed by luciferase/3′-UTR reporter assay; ** unspecified target.

**Table 2:** MicroRNAs pattern in GC cells and potential treatments.

**If These miRNAs Are *UPREGULATED***	**Consider a Potential Treatment with**
miR-200bc/429	VCR, CDDP, VP-16, ADR
miR-181b	
miR-497	
miR-15b	
miR-16	
miR-508-5p	VCR, CDDP, 5-Fu,
miR-129-5p	
miR-23b-3p	
miR-503	
**If these miRNAs are *DOWNREGULATED***	**Consider a potential treatment with**
miR-19a/b	CDDP
miR-106a	
miR-21	
miR-20a	
miR-223	

## Abbreviations

ABCB1: ATP-binding cassette, sub-family B, member 1
IGF1R: insulin-like growth factor 1 receptor
IRS1: insulin receptor substrate 1
ZNRD1: zinc ribbon domain-containing
PTEN: phosphatase and tension homolog
BCL2: B-cell lymphoma 2
XIAP: X-linked inhibitor of apoptosis protein
RUNX3: runt-related transcription factor 3
SMO: Smoothened
MAPT: microtubule associated protein tau
HMGA2: high-mobility group AT-hook 2
IRF1: interferon regulator factor 1
VCR: vincristine
CDDP: cisplatin
ADR: adriamycin
VP-16: etoposide
5-Fu: 5-fluoruracil
DOX: doxorubicin
DTX: docetaxel
GEM: GEM
PTX: paclitaxel
L-OHP: oxaliplatin
